# Dose-Summation–Guided Re-irradiation Planning for Brain Metastases: Cross-Platform Verification Between Brainlab Elements and GammaPlan

**DOI:** 10.7759/cureus.98296

**Published:** 2025-12-02

**Authors:** Shigeo Matsunaga, Takashi Shuto, Jo Sasame

**Affiliations:** 1 Department of Neurosurgery and Stereotactic Radiotherapy Center, Yokohama Rosai Hospital, Yokohama, JPN

**Keywords:** brainlab elements, brain metastases, cumulative dose, dose summation, eqd2, gamma knife, radiation necrosis, re-irradiation, stereotactic radiosurgery, v12

## Abstract

We report a practical, cross-platform workflow for cumulative-dose-aware re-irradiation in brain metastases. Before RT-DOSE is imported across planning systems (Brainlab Elements and/or GammaPlan), previously irradiated foci are treated prospectively as organs-at-risk during optimization, and the summed dose is visualized to avoid hotspots while preserving coverage. In a verification case planned without dose summation, a non-target previously irradiated focus ultimately developed radiation necrosis; post-hoc summation showed that explicit constraints could have reduced cumulative exposure. In a second case prospectively planned with summation, target coverage was maintained while decreasing the unintended dose to a non-target previously treated focus, with subsequent control and no new toxicity. This cross-platform approach is feasible and clinically actionable for balancing efficacy and safety during repeat stereotactic radiosurgery near prior high-dose regions.

## Introduction

As survival has improved with modern systemic therapies and advanced imaging detects ever smaller intracranial lesions, the incidence of brain metastases continues to rise [1,2]. Consequently, repeated stereotactic radiosurgery (SRS) for new or recurrent brain metastases has become common in clinical practice [3,4]. Large multi-institutional and large-cohort data, including the JLGK0901 trial and companion series, demonstrate that SRS alone is safe and effective for patients harboring multiple brain metastases, even when five or more (and up to ≥10) lesions are treated [5-8]. Randomized evidence suggests that, for patients with one to three metastases, SRS alone limits cognitive deterioration compared with whole-brain radiotherapy without compromising overall survival, underscoring the value of focal approaches [3,9]. However, when a new target abuts a previously irradiated focus, ignoring historical dose risks, unrecognized hotspots, and iatrogenic injury. We implemented a cross-platform strategy that (i) imports prior RT-DOSE, (ii) sums dose across courses and platforms, and (iii) prospectively treats previously irradiated foci as organs-at-risk (OARs) during optimization; this design was informed by accumulating evidence that cumulative dose to the brain and critical structures, including overlap volumes across radiation courses, predicts toxicity and guides safe re-irradiation in intracranial tumors [10-15], that normal-brain dose-volume metrics (e.g., V12) correlate with symptomatic radionecrosis [16,17], and that RTOG 90-05 single-fraction dose-escalation limits provide practical guardrails for retreatment [8,18-20]. We then verified its clinical implications in two representative cases.

## Technical report

Platforms and imaging

Planning was performed with Brainlab Elements (v4.0; Brainlab, Munich) and GammaPlan (v11.4.2; Elekta, Stockholm). Datasets included non-contrast CT and contrast-enhanced T1-weighted MRI (1-mm slices) plus T2-weighted MRI (2-mm, no gap).

Cross-platform transfer and dose summation

Prior RT-DOSE (DICOM RT) was transferred between systems, rigidly co-registered to the current dataset, and visualized as cumulative isodose volumes. When summation was not natively available, RT-DOSE from the other platform was imported for verification.

Immobilization and delivery

Gamma Knife SRS used the Leksell Gamma Knife Icon (frame or mask). LINAC-based SRS used TrueBeam STx/Novalis with thermoplastic mask immobilization and multiple dynamic conformal arcs (typical prescription ~70% isodose), with fractionation individualized.

Planning strategy (previous foci treated as OARs)

Planning was performed with prior RT-DOSE imported and rigidly co-registered to the current dataset. Previously irradiated foci were contoured and treated as OARs during optimization. For each re-irradiation, we generated and compared two plans: (1) an unconstrained plan that did not apply limits to the prior foci, and (2) a summation-guided plan that prospectively constrained the prior foci while maintaining planning target volume (PTV) coverage. Plan optimization prioritized target coverage and conformity while explicitly minimizing the cumulative dose to previously irradiated foci based on the summed (initial + re-irradiation) dose distribution [4,10]. In practice, we targeted cumulative Dmax ≤ ~65-70 Gy and cumulative Dmean ≤ ~55-60 Gy to the prior focus when feasible and monitored normal-brain V12_cum alongside standard target metrics (e.g., PTV coverage/D95) and isodose line (IDL) selection (typically ~70%). These values were used as planning guardrails rather than absolute cutoffs, informed by single-fraction dose-escalation limits (RTOG 90-05) and by reports linking normal-brain V12 to symptomatic radionecrosis after SRS [16-18] (with contemporary data in modern systemic therapy settings) [10]. Cumulative maxima/means within prior foci and PTV coverage were recorded for both plans, and the final plan was selected to preserve target coverage while reducing exposure to previously irradiated non-target foci.

Case presentation

Case 1: Delivered Without Dose Summation and Verification Added Later

**Figure 1:**
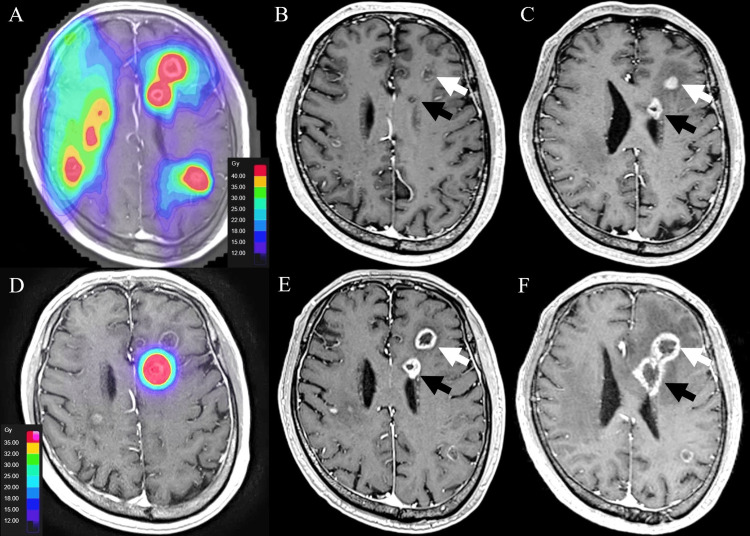
Case 1 (delivered without dose summation) (A) Initial Brainlab Elements plan (Multiple Brain Mets SRS): 40 Gy in 10 fractions prescribed to the 70% isodose, with the composite RT-dose distribution superimposed on the baseline contrast-enhanced T1-weighted MR image (the underlying tumor margins remain faintly visible). (B, C) Baseline contrast-enhanced T1-weighted MR images without RT-dose overlay demonstrating the left frontal index metastasis (white arrow) and an adjacent previously irradiated focus (black arrow); panel B (four-month follow-up) shows partial regression and panel C (eight-month follow-up) shows subsequent regrowth of the index lesion. (D) Brainlab Elements re-irradiation: 35 Gy in five fractions. (E) Early (three-month) post–re-irradiation follow-up demonstrating a subtle decrease in lesion size with thinning of the enhancing rim and enlargement of the central low-signal area (white arrow), with minimal change in the adjacent previously treated focus (black arrow). (F) Six-month follow-up showing enlargement of both the re-irradiated target and the adjacent previously treated, non-target left frontal focus (white and black arrows), findings consistent with radiation necrosis.

A man in his 60s with ALK-rearranged lung adenocarcinoma presented with left hemiparesis. Twelve metastases were treated using Brainlab Elements Multiple Brain Mets SRS (40 Gy/10 fx to the 70% isodose; eight arcs) (Figure 1A).

Lesions regressed at four months (Figure 1B). At eight months, a left frontal lesion regrew (Figure 1C) and was re-irradiated (35 Gy/5 fx; six arcs) as the sole target (Figure 1D); the adjacent previously treated left frontal focus-non-target at re-irradiation-later enlarged, consistent with radiation necrosis (RN) (Figures 1E, 1F).

**Figure 2:**
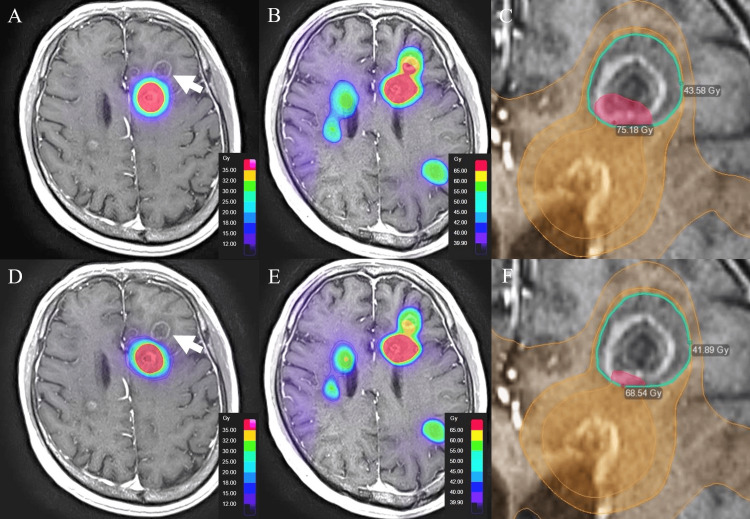
Case 1 verification in Brainlab Elements using dose summation (A) Plan 1 without constraints to the non-target left frontal focus (white arrow). (B) Summed dose for the initial and re-irradiation plans. (C) Cumulative metrics within the non-target focus for Plan 1 (max 75.18 Gy; mean 58.64 Gy). (D) Plan 2 with constraints to the non-target focus. (E) Summed dose for Plan 2. (F) Cumulative metrics within the non-target focus for Plan 2 (max 68.54 Gy; mean 56.23 Gy).

Brainlab Elements verification: Plan 1 (no constraint to the non-target left frontal focus) followed by dose summation yielded cumulative max 75.18 Gy and mean 58.64 Gy in that focus (Figures 2A-2C).

Plan 2 (explicit constraints to that focus) reduced the cumulative exposure to max 68.54 Gy and mean 56.23 Gy (Figures 2D-2F).

**Figure 3:**
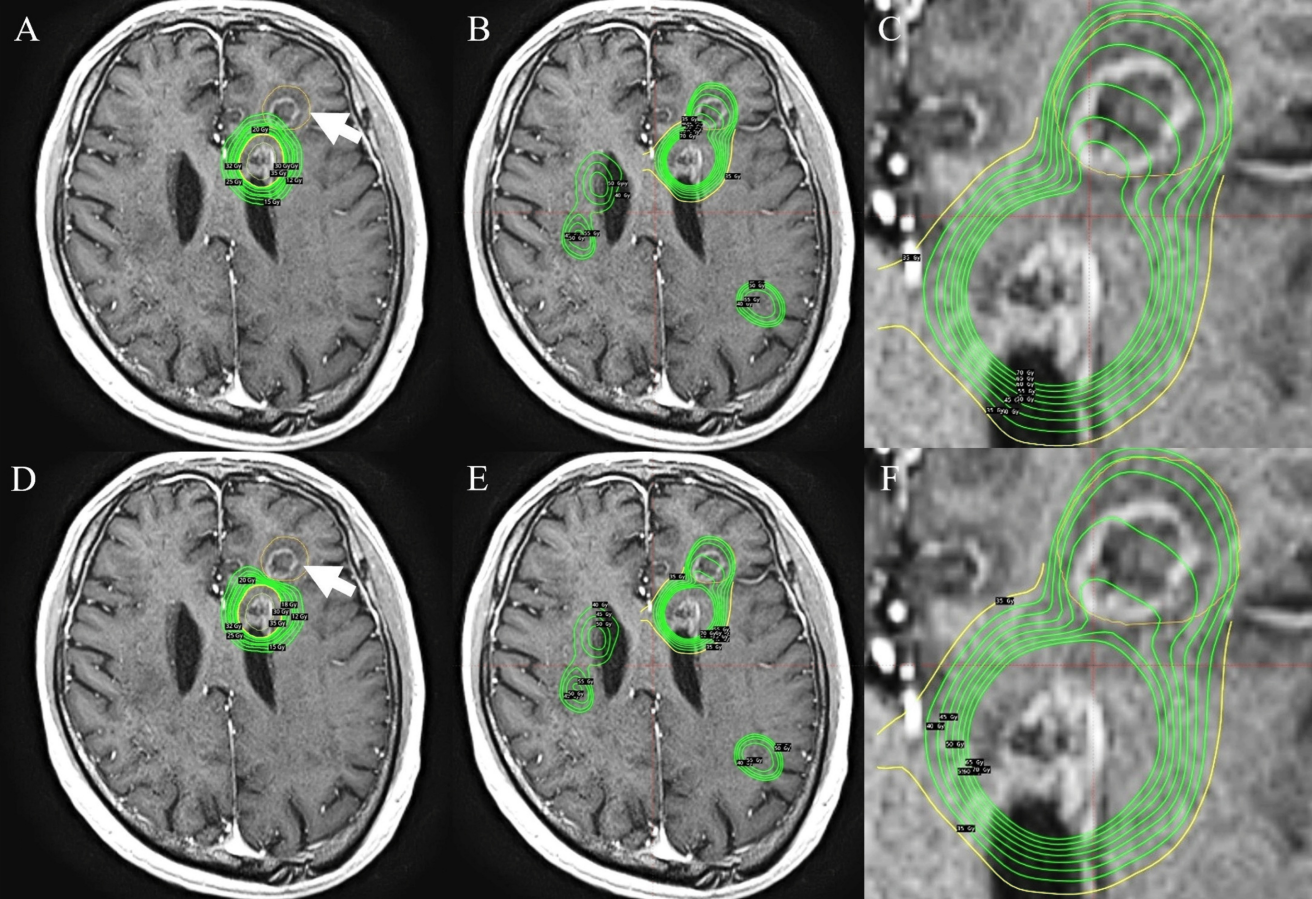
Case 1 verification in GammaPlan using dose summation (A) Plan 3 without constraints to the non-target left frontal focus (white arrow). (B) Summed dose for the initial and re-irradiation plans. (C) Cumulative metrics within the non-target focus for Plan 3 (max 75.4 Gy; mean 57.9 Gy). (D) Plan 4 with constraints to the non-target focus. (E) Summed dose for Plan 4. (F) Cumulative metrics within the non-target focus for Plan 4 (max 67.1 Gy; mean 55.9 Gy).

GammaPlan verification: Importing the initial Elements RT-DOSE, Plan 3 (no constraint) yielded cumulative max 75.4 Gy and mean 57.9 Gy (Figures 3A-3C); Plan 4 (with constraints) reduced to max 67.1 Gy and mean 55.9 Gy (Figures 3D-3F).

Although platform-specific differences were small, both showed consistent reductions with summation-guided constraints. Had Plan 2 (Brainlab Elements verification) (Figure 2D-F)/Plan 4 (GammaPlan verification) (Figure 3D-F) been delivered clinically, complete prevention of radionecrosis cannot be guaranteed, but severity might have been mitigated.

Case 2: Planned and Delivered With Dose Summation

**Figure 4:**
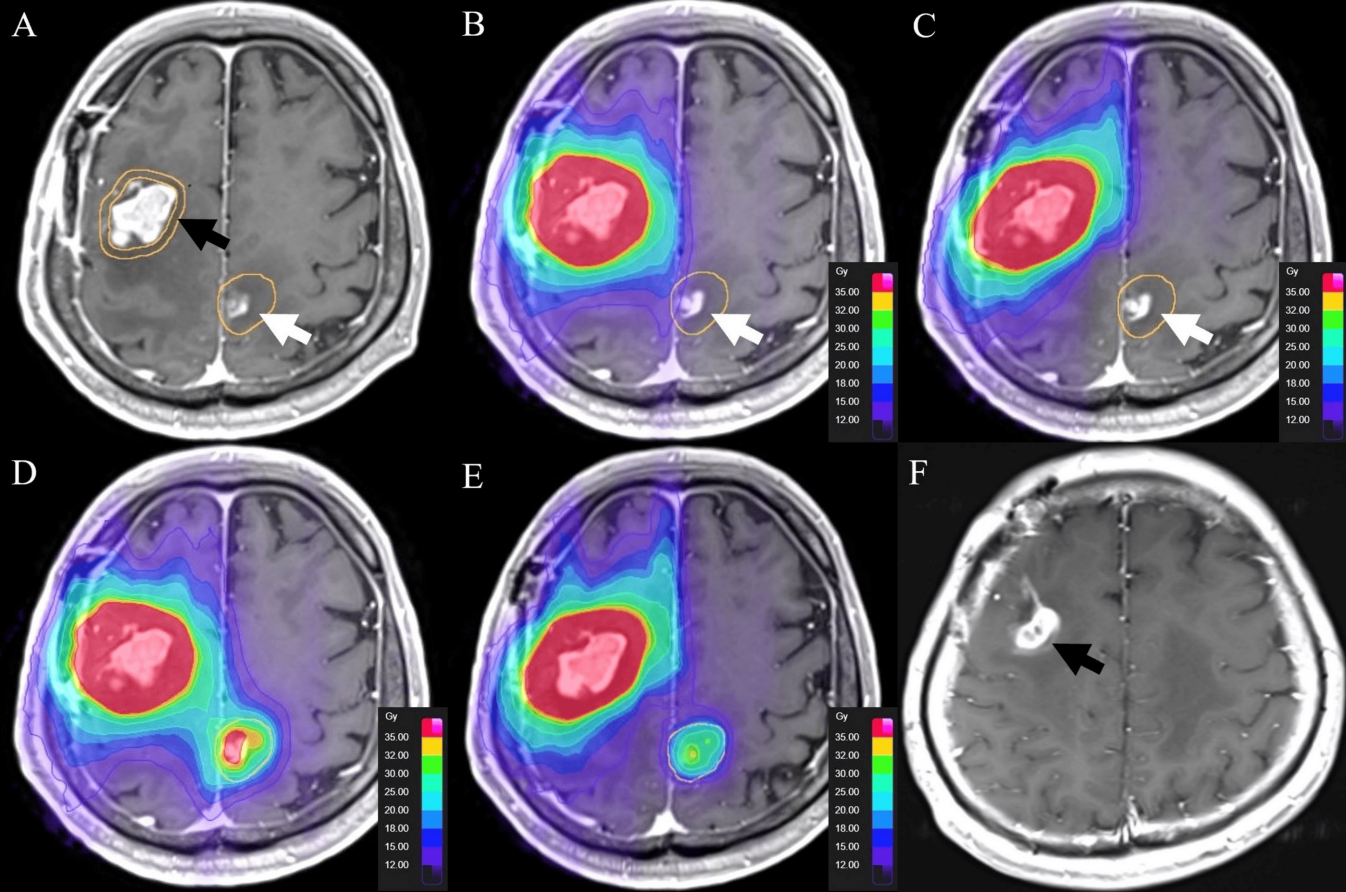
Case 2 (planned and delivered with dose summation) (A) Recurrence at the right frontal postoperative cavity (black arrow). (B) Brainlab Elements Plan 1 without constraints to the previously treated left parietal focus (white arrow). (C) Plan 2 with explicit constraints to that non-target focus. (D and E) Summed dose distributions for Plans 1 and 2. (F) Six-month decrease of the right frontal target with near-resolution and continued control of the previously treated left parietal focus.

A man in his 50s with gastroesophageal junction cancer developed multiple brain metastases, including a right frontal lesion requiring surgery and 14 additional small lesions treated with Gamma Knife SRS (single fraction 20 Gy to 51-90% IDL). The right frontal cavity recurred (Figure 4A).

Re-irradiation was planned in Elements (10 arcs; 35 Gy/5 fx at 70% IDL). Prior GammaPlan RT-DOSE was imported. Plan 1 (no constraint to a non-target left parietal focus previously treated by GK) yielded higher non-target dose on summation (Figure 4B), whereas Plan 2 with explicit constraints reduced the non-target exposure while maintaining PTV coverage (Figure 4C). The summed dose distributions, combining the initial GammaPlan RT-DOSE with the re-irradiation RT-DOSE from Elements, for Plan 1 and Plan 2 are shown in Figure 4D and Figure 4E, respectively. At six months, the right frontal target decreased and the previously treated left parietal focus nearly disappeared while remaining controlled (Figure 4F).

## Discussion

Summation-guided planning operationalizes a simple principle: visualize the true cumulative dose and treat previously irradiated foci as OARs while protecting the current target. Without summation, imposing constraints based on geometric surrogates (e.g., prior GTV/PTV shells) can distort both peripheral and internal high-dose regions and may compromise coverage. Summation makes these trade-offs explicit, enabling an informed choice between constrained and unconstrained solutions.

In our experience, Case 1 was re-irradiated in routine practice without dose summation, and the adjacent, previously irradiated non-target focus ultimately developed RN. In contrast, verification performed in both Brainlab Elements and GammaPlan demonstrated that when the previously treated non-target focus was prospectively constrained as an OAR, the cumulative dose (max/mean) to that focus was consistently reduced across platforms. In Case 2, summation was used prospectively during planning, which maintained PTV coverage while lowering exposure to the non-target previously irradiated focus.

Summation-guided planning primarily advances how retreatment decisions are made: cumulative dose is rendered explicit, previously treated foci are constrained like OARs, and target coverage is maintained with fewer unexpected effects on adjacent high-dose regions, an approach that is consistent with emerging data on the clinical relevance of cumulative-dose evaluation in repeat intracranial irradiation [9,10] and with reports linking normal-brain dose-volume metrics such as V12 to symptomatic radionecrosis after SRS [11,16] (including contemporary series evaluating V12 and adverse radiation effects in modern systemic therapy contexts [19]). Beyond V12, prior studies have explored additional dose, volume or biologically adjusted metrics, V10-V14 and Dmax [16,17], as well as BED/EQD2 and V12_eq [10,11], as correlates of symptomatic radionecrosis. Taken together with the single-fraction limits from RTOG 90-05 and the association between normal-brain V12 and adverse radiation effects, these cumulative constraints serve as planning guardrails rather than absolute cutoffs, helping balance target coverage against injury risk during retreatment. Our two cases, verified across Brainlab Elements and GammaPlan, illustrate cross-platform, consistent reductions in exposure to non-target prior foci when summation-based constraints are applied, while preserving PTV coverage. In day-to-day practice, conventional single-fraction dose-escalation limits (RTOG 90-05) can serve as pragmatic guardrails within this workflow [18].

Given the retrospective design and the inclusion of only two patients, no definitive conclusions regarding safety or clinical efficacy can be drawn from this report. Rather, our aim is to illustrate a technically simple, readily adoptable workflow for repeat intracranial SRS near previously high-dose regions and to highlight how it can be implemented in daily practice. Future work, ideally in prospective, multi-institutional cohorts, is needed to validate whether such summation-guided planning actually improves toxicity and tumor control outcomes and to explore thresholding strategies (e.g., V12-derived or EQD2/BED-based limits) that can be operationalized within planning systems.

## Conclusions

Summation-guided re-irradiation primarily advances how retreatment decisions are made: cumulative dose is rendered explicit, previously treated foci are prospectively constrained as OARs, and target coverage is preserved while minimizing unexpected dose escalation in adjacent high-dose regions. In our two cases, cross-platform verification in Brainlab Elements and GammaPlan showed consistent reductions in exposure to non-target prior foci when summation-based constraints were applied, without compromising PTV coverage. Given its technical feasibility and clinical clarity, we recommend incorporating cumulative-dose summation as a routine checkpoint for repeat intracranial SRS near prior high-dose areas, while acknowledging that broader prospective validation and operational thresholding (e.g., V12-derived or EQD2/BED-based limits) are warranted.

## References

[1] Nayak L, Lee EQ, Wen PY (2012). Epidemiology of brain metastases. Curr Oncol Rep.

[2] Mitchell DK, Kwon HJ, Kubica PA, Huff WX, O'Regan R, Dey M (2022). Brain metastases: an update on the multi-disciplinary approach of clinical management. Neurochirurgie.

[3] Aoyama H, Shirato H, Tago M (2006). Stereotactic radiosurgery plus whole-brain radiation therapy vs stereotactic radiosurgery alone for treatment of brain metastases: a randomized controlled trial. JAMA.

[4] Yuan J, Lee R, Dusenbery KE, Lee CK, Mathew DC, Sperduto PW, Watanabe Y (2018). Cumulative doses to brain and other critical structures after multisession Gamma Knife stereotactic radiosurgery for treatment of multiple metastatic tumors. Front Oncol.

[5] Yamamoto M, Serizawa T, Shuto T (2014). Stereotactic radiosurgery for patients with multiple brain metastases (JLGK0901): a multi-institutional prospective observational study. Lancet Oncol.

[6] Yamamoto M, Serizawa T, Higuchi Y (2017). A multi-institutional prospective observational study of stereotactic radiosurgery for patients with multiple brain metastases (JLGK0901 study update): irradiation-related complications and long-term maintenance of mini-mental state examination scores. Int J Radiat Oncol Biol Phys.

[7] Yamamoto M, Kawabe T, Sato Y (2013). A case-matched study of stereotactic radiosurgery for patients with multiple brain metastases: comparing treatment results for 1-4 vs ≥ 5 tumors: clinical article. J Neurosurg.

[8] Yamamoto M, Kawabe T, Sato Y, Higuchi Y, Nariai T, Watanabe S, Kasuya H (2014). Stereotactic radiosurgery for patients with multiple brain metastases: a case-matched study comparing treatment results for patients with 2-9 versus 10 or more tumors. J Neurosurg.

[9] Brown PD, Jaeckle K, Ballman KV (2016). Effect of radiosurgery alone vs radiosurgery with whole brain radiotherapy on cognitive function in patients with 1 to 3 brain metastases: a randomized clinical trial. JAMA.

[10] Oh E, Garg S, Liu Y (2018). Identification and functional characterization of anti-metastasis and anti-angiogenic activities of triethylene glycol derivatives. Front Oncol.

[11] Kuntz L, Le Fèvre C, Jarnet D (2023). Acute toxicities and cumulative dose to the brain of repeated sessions of stereotactic radiotherapy (SRT) for brain metastases: a retrospective study of 184 patients. Radiat Oncol.

[12] Touati R, Bourbonne V, Dissaux G (2023). Re-irradiation by stereotactic radiotherapy of brain metastases in the case of local recurrence. Cancers (Basel).

[13] Maranzano E, Terenzi S, Anselmo P (2019). A prospective phase II trial on reirradiation of brain metastases with radiosurgery. Clin Transl Radiat Oncol.

[14] Rades D, Simone CB 2nd, Wong HC, Chow E, Lee SF, Johnstone PA (2024). Reirradiation of metastases of the central nervous system: part 1-brain metastasis. Ann Palliat Med.

[15] Kato T, Hasegawa T, Kuwabara K (2025). Re-irradiation followed by resection for recurrent brain metastases after initial stereotactic radiosurgery: illustrative cases. J Neurosurg Case Lessons.

[16] Minniti G, Clarke E, Lanzetta G (2011). Stereotactic radiosurgery for brain metastases: analysis of outcome and risk of brain radionecrosis. Radiat Oncol.

[17] Blonigen BJ, Steinmetz RD, Levin L, Lamba MA, Warnick RE, Breneman JC (2010). Irradiated volume as a predictor of brain radionecrosis after linear accelerator stereotactic radiosurgery. Int J Radiat Oncol Biol Phys.

[18] Shaw E, Scott C, Souhami L (2000). Single dose radiosurgical treatment of recurrent previously irradiated primary brain tumors and brain metastases: final report of RTOG protocol 90-05. Int J Radiat Oncol Biol Phys.

[19] Helis CA, Hughes RT, Glenn CW (2020). Predictors of adverse radiation effect in brain metastasis patients treated with stereotactic radiosurgery and immune checkpoint inhibitors. Int J Radiat Oncol Biol Phys.

[20] Fritz C, Borsky K, Stark LS (2018). Repeated courses of radiosurgery for new brain metastases to defer whole brain radiotherapy: feasibility and outcomes with validation of brain metastasis velocity. Front Oncol.

